# Effects of Argonaute on Gene Expression in *Thermus thermophilus*

**DOI:** 10.1371/journal.pone.0124880

**Published:** 2015-04-22

**Authors:** Daan C. Swarts, Jasper J. Koehorst, Edze R. Westra, Peter J. Schaap, John van der Oost

**Affiliations:** 1 Laboratory of Microbiology, Wageningen University, Wageningen, The Netherlands; 2 Laboratory of Systems and Synthetic Biology, Wageningen University, Wageningen, The Netherlands; Max-Planck-Institute for Terrestrial Microbiology, GERMANY

## Abstract

**Background:**

Eukaryotic Argonaute proteins mediate RNA-guided RNA interference, allowing both regulation of host gene expression and defense against invading mobile genetic elements. Recently, it has become evident that prokaryotic Argonaute homologs mediate DNA-guided DNA interference, and play a role in host defense. Argonaute of the bacterium *Thermus thermophilus* (*Tt*Ago) targets invading plasmid DNA during and after transformation. Using small interfering DNA guides, *Tt*Ago can cleave single and double stranded DNAs. Although *Tt*Ago additionally has been demonstrated to cleave RNA targets complementary to its DNA guide *in vitro*, RNA targeting by *Tt*Ago has not been demonstrated *in vivo*.

**Methods:**

To investigate if *Tt*Ago also has the potential to control RNA levels, we analyzed RNA-seq data derived from cultures of four *T*. *thermophilus* strain HB27 variants: wild type, *Tt*Ago knockout (Δ*ago*), and either strain transformed with a plasmid. Additionally we determined the effect of *Tt*Ago on expression of plasmid-encoded RNA and plasmid DNA levels.

**Results:**

In the absence of exogenous DNA (plasmid), *Tt*Ago presence or absence had no effect on gene expression levels. When plasmid DNA is present, *Tt*Ago reduces plasmid DNA levels 4-fold, and a corresponding reduction of plasmid gene transcript levels was observed. We therefore conclude that *Tt*Ago interferes with plasmid DNA, but not with plasmid-encoded RNA. Interestingly, *Tt*Ago presence stimulates expression of specific endogenous genes, but only when exogenous plasmid DNA was present. Specifically, the presence of *Tt*Ago directly or indirectly stimulates expression of CRISPR loci and associated genes, some of which are involved in CRISPR adaptation. This suggests that *Tt*Ago-mediated interference with plasmid DNA stimulates CRISPR adaptation.

## Introduction

Argonaute proteins (Agos) have long been known as key players in eukaryotic RNA interference (RNAi) pathways, in which eukaryotic Ago (eAgo) uses a small single-stranded (ss)RNA guide to target ssRNA molecules (reviewed in [1–3]). While many RNAi pathways regulate host gene expression by targeting mRNAs, some RNAi pathways are involved in host defense (reviewed in [4–6]). In these pathways, Agos interfere with RNA transcripts from viruses or transposons, or with RNA viruses directly.

Prokaryotes also encode Agos (pAgos), but none of the additional proteins involved in canonical RNAi pathways [7–10]. Recently, it has become clear that pAgos are involved in mediating host defense, but in contrast to eAgos, they target DNA rather than RNA [11,12]. One of the best studied pAgos is that of *Thermus thermophilus* (*Tt*Ago), which has been characterized structurally and biochemically [12–16]. *T*. *thermophilus* is a gram-negative thermophilic bacterium that is used as model organism for genetic transformation, biotechnological applications and structural biology. *T*. *thermophilus* strain HB27 has a 1.9 Mb chromosome encoding 1988 genes (with TTC# tag) and harbors a 232 Kb mega-plasmid designated pTT27, encoding 230 genes (with TT_P# tag).

In contrast to RNA-guided eAgos, *Tt*Ago has been demonstrated to utilize DNA guides in order to cleave single stranded RNA (ssRNA), single stranded DNA (ssDNA) and double stranded DNA (dsDNA) targets *in vitro* [12–16]. This allows *Tt*Ago to directly interfere with invading DNAs, lowering plasmid transformation efficiencies and intracellular plasmid content [12,17]. As *Tt*Ago preferentially acquires guides from plasmid DNA [12], and it is able to cleave RNA targets *in vitro*, it was predicted that *Tt*Ago also interferes with plasmid transcripts [12]. This would suggest a dual-function of *Tt*Ago, both in defense and in gene regulation, which is akin to eAgos [1–3] and prokaryotic CRISPR-Cas [18]. However, gene expression of *T*. *thermophilus* has not yet been investigated in strains in which invading DNA in the form of a plasmid was present.

Here, we describe the analysis of a new RNA-seq dataset derived from *T*. *thermophilus* strains HB27 and HB27Δ*ago* harboring plasmid pMKPnqosGFP [19]. Although the presence of *Tt*Ago or plasmid DNA itself does not strongly affect gene expression, the presence of both results in decreased quantities of plasmid-encoded RNA transcripts and increased expression of specific genomic genes.

## Results and Discussion

**Fig 1:**
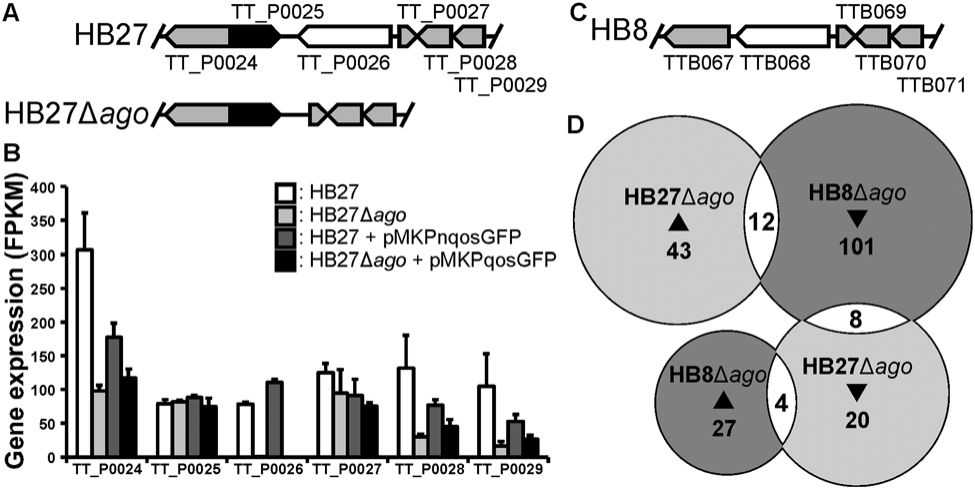
Δ*ago* result in stochastic changes in gene expression in *T*. *thermophilus* strains. A, Schematic representation of the gene regions encoding *Tt*Ago (TT_P0026) of *T*. *thermophilus* strain HB27 and HB27Δ*ago*. B, Schematic representation of the gene regions encoding TTB068 in *T*. *thermophilus* strain HB8. As no information on how the HB8 *ago* knockout was generated is available [21], HB8Δ*ago* is not displayed. HB8 genes colored grey and white are homologous to the HB27 genes indicated in Fig 1A. C, Expression of genes located near *ago* (TT_P0026) on the genome. Expression values are given in Fragments Per Kilobase of exon per Million fragments mapped (FPKM). D, Overlap in >2-fold up-regulated (▲) and >2-fold down-regulated (▼) homologous genes in HB27Δ*ago* relative to HB27, and HB8Δ*ago* relative to HB8.

We included previously obtained RNA-seq data from HB27 and HB27Δ*ago* [12] in our analysis in order to compare them with the new data from HB27 + plasmid (HB27+P) and HB27Δ*ago* + plasmid (HB27Δ*ago*+P). The latter two strains were grown in medium containing kanamycin, selecting for plasmid maintenance. For each condition, RNA from biological triplicates was purified, sequenced and mapped, and for each gene the abundance was calculated as Fragments Per Kilobase of exon per Million fragments mapped (FPKM). RNA levels are considered to be changed significantly when the FPKM value of a set of biological triplicates differed from the FPKM value of another set of biological triplicates with *P*<0.05. We considered changes in RNA levels biologically relevant if FPKM averages of biological triplicates differed at least >4-fold from FPKM averages of another set of biological triplicates, while smaller changes were considered stochastic. RNA was purified from triplicate log phase (OD_600 nm_ of 0.5) cultures HB27, HB27Δ*ago*, HB27+P and HB27Δ*ago*+P (Fig 1A). Using Prodigal 2.6 [20], 35 new open reading frames were identified of which 22 were located on the HB27 chromosome (tagged TTCX01-TTCX22) and 13 on the mega-plasmid pTT27 (TTPX01-TTPX13). Of these new genes, 15 encode proteins of which the function can be predicted based on (partial) homology to other proteins. Furthermore, 16 encode proteins that show (partial) similarity to hypothetical proteins, whereas four encode proteins that share no significant similarity to other proteins in the current NCBI database. The open reading frames and predicted functions of the proteins they encode are listed in S1 Table.

### The absence of *Tt*A*go* results in small stochastic changes in *T*. *thermophilus* gene expression

The *ago* knockout in *T*. *thermophilus* strain HB27 has previously been demonstrated to result in small pleiotropic changes in gene expression (<4-fold change for most genes) [12], and this was confirmed in our new analyses of the same dataset (S2 Table). Stochastic changes in gene expression include 59 genes which are >2-fold up-regulated and 35 genes which are >2-fold down-regulated in HB27Δ*ago* compared to HB27 (S2 Table). Besides these small differences, >4-fold change in expression was observed for specific genes (S2 Table). As expected, we observe no expression of the gene encoding *Tt*Ago (TT_P0026) in Δ*ago* strains, and low levels of *Tt*Ago expression in wild type strains (FPKM<150; Fig 1B). In agreement with this observation, evidence for expression of (strep(II)-tagged) *Tt*Ago protein encoded by the knock-in gene at the same genomic location has previously been demonstrated [12]. The *ago* knockout resulted in 3 to 6-fold lower RNA levels mapped against genes located near and on the same strand as *ago* (Fig 1B). These changes are most likely polar effects caused by *ago* deletion. RNA mapped against two other genes is lowered 3-fold and 5-fold in HB27Δ*ago*: TTC1213 (1-pyrroline-5-carboxylate dehydrogenase) and TTC1241 (predicted acyl-amino acid-releasing enzyme). These genes, as well as the genes located near *ago* on the genome, are also down-regulated in HB27Δ*ago*+P compared to HB27+P. In addition, a predicted operon encoding a branched-chain amino acid transport system (TTC0333-TTC0343) appears up-regulated (3 to 5-fold increase in RNA levels) in HB27Δ*ago* compared to HB27. This operon encodes a system homologous to the Liv ABC transporter system, which transports the amino acids leucine, isoleucine, valine, threonine and alanine in an ATP dependent manner. The same set of genes is only moderately up-regulated (most genes <2-fold change) in HB27+P compared to HB27Δ*ago*+P. A functional link between these genes and the *ago* knockout is not obvious. The levels of these RNAs are affected in both HB27Δ*ago* and HB27Δ*ago*+P, suggesting that *Tt*Ago affects these RNA levels directly or indirectly.

### Comparison of *T*. *thermophilus* HB8Δ*ago* and HB27Δ*ago*

A recent publication describes the differences in RNA expression between *T*. *thermophilus* strains HB8 (Fig 1C) and HB8Δ*ago* [21]. The chromosomes of *T*. *thermophilus* strains HB8 and HB27 are highly conserved, while their mega-plasmids pTT8 and pTT27, which encode *ago* and most CRISPR-Cas related genes, show a higher degree of divergence [22]. RNA was purified from log-phase cultures in both studies, but the growth medium used for HB8 cultivation [21] is slightly different from the medium we used for HB27 cultivation (S3 Table). We compared the genes from HB27Δ*ago* and HB8Δ*ago* of which corresponding RNA levels changed >2-fold compared to the corresponding wild type strains. We found no clear correlation between the affected genes in both strains (Fig 1D and S3 Table). None of the genes of which expression changed >4-fold in HB8Δ*ago* were found to be differentially expressed in HB27Δ*ago* (S3 Table).

Given that Ago proteins interact with guides to bind specific complementary targets [10,23], it would be expected that *Tt*Ago strongly affects levels of specific RNAs. As chromosomes of both HB8 and HB27 *Tt*Ago are very similar, and specific RNA levels changed in HB8Δ*ago* and HB27Δ*ago* vary greatly, it seems unlikely that *Tt*Ago targets specific RNAs. Instead, our analysis suggests that observed differences in RNA levels are stochastic, and thus unlikely to be caused by guided *Tt*Ago activity. The observation that *Tt*Ago does not influence the transcription of genes involved in competence or host defense, suggests that *Tt*Ago only interferes with the invading DNA directly. In a recent study the competence of HB27 and HB27Δ*ago* has been compared during natural transformation experiments and during cell-to-cell conjugation experiments with genomic *T*. *thermophilus* DNA. It was found that *Tt*Ago does interfere with natural transformation, but not with cell-to-cell conjugation [17]. As the genes required for natural transformation are also essential for cell-to-cell conjugation, this excludes a possible indirect effect of *Tt*Ago via regulation of expression competence genes.

### Presence of plasmid DNA results in changes in gene expression only if *Tt*Ago is present

To investigate the effect of the presence of plasmid DNA on gene expression, we compared RNA isolated from HB27Δ*ago* to that from HB27Δ*ago*+P. No significant (*P*<0.05) >4-fold changes in RNA levels were observed. Furthermore, the presence of plasmid DNA did not result in significant (*P*<0.05) >2-fold change in expression of host-defense genes (S2 Table). Combined, these data suggest that presence of invading nucleic acids in the form of plasmid DNA does not result in differentiated gene expression in HB27Δ*ago*. This contrasts with the presence of another invader, lytic phage ϕYS40 [24] in HB8, which results in up-regulation of a plethora of host defense genes. These genes encode (amongst others) *Tt*Ago, the *T*. *thermophilus* Type I-E (not encoded in HB27), Type III-A and Type III-B CRISPR-Cas systems, as well as multiple other Cas genes scattered over the HB8 genome [24]. In summary, although phage infection triggers host defense response pathways in *T*. *thermophilus*, the presence and replication of plasmid DNA does not trigger host defense responses. This is presumably because defense pathways are costly to induce [25], and are most beneficial in the context of parasitic infections [26], such as by lytic phages. In contrast, plasmids are far less detrimental to the host and often confer a fitness benefit [27], making it unnecessary to induce these pathways during plasmid invasion.

**Fig 2:**
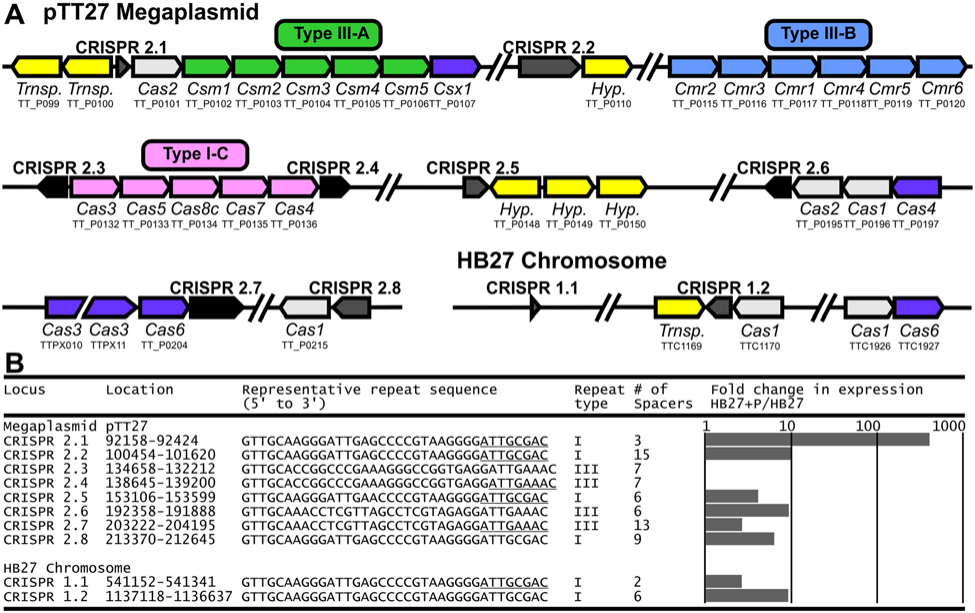
CRISPR loci and cas genes encoded by *T*. *thermophilus* HB27. A, Schematic representation of CRISPR loci and *cas* genes encoded on mega-plasmid pTT27 and the *T*. *thermophilus* HB27 chromosome. Encoded protein and KEGG annotation are given below each gene. Note that size of illustrated genes do not correspond to their actual size. CRISPR loci with type I and III repeats are colored gray and black, respectively. Repeat types are based on [28] and should not be confused with CRISPR-Cas Types [29]. Transp: Transposase. Hyp: Hypothetical protein. B, Characteristics of CRISPR loci encoded by *T*. *thermophilus* HB27. Fold change in CRISPR RNA levels is shown for HB27+P compared to HB27.

In contrast, when comparing WT (Ago-encoding) strains with and without plasmid DNA, we observed significant (*P*>0.02) >4-fold increase of RNA levels mapped to specific genes (HB27+P compared to HB27; Table 1, S2 Table). Corresponding genes, difference in RNA levels, motifs and predicted functions of the proteins they encode are listed in Table 1 and Fig 2.

**Table 1:** Genes differentially expressed in HB27+P compared to HB27.

Gene	Fold change*	Motif(s), derived from KEGG	Function (predicted)	Located near CRISPR locus?
TT_P0099	4.3	DDE 3	**Transposase	Upstream CRISPR 2.1 (reverse orientation)
TT_P0101	27.9	CRISPR Cas2	Cas2; involved in CRISPR-adaptation	Directly downstream CRISPR 2.1
TT_P0110	8.1	DUF1887	Hypothetical protein, **Csx1	Directly downstream CRISPR 2.2
TT_P0149	27.1	C-terminal AAA-associated	Hypothetical protein	Directly downstream CRISPR 2.5
TT_P0150	4.0	ABM, DUF1330, Dehydratase-heme	Hypothetical protein	Directly downstream CRISPR 2.5
TT_P0211	6.6	AAA 14, DUF4143, HTH motifs, MopB	**ATPase, **Cas1, **Transposase, **Transcription regulation	Downstream CRISPR 2.7 (reverse orientation)
TTPX09	5.9	-	Putative gene	Located in CRISPR 2.6
TTPX12	6.6	-	Putative gene	(Far) downstream CRISPR 2.7 (reverse orientation)
TTC0310	4.2	PAPS reduct	**Phosphoadenosine phosphosulfate reductase	No
TTC0311	4.3	NAD binding 7, CysG dimeriser, Sirohm synth M	**Uroporphyrin-III C-methyltransferase	No
TTC0399	4.1	-	Hypothetical protein	No
TTC1169	12.6	DEDD Tnp IS110, Transposase 20, Helix-Hairpin-Helix (HHH)	**Transposase, **DNA binding	Directly downstream CRISPR 1.2, (reverse orientation)

*: Fold-change increase in RNA levels in HB27+P compared to HB27. For all changes *P*<0.02.

**: Function predicted based on domains and similarity to other genes.

There seems to be no clear link between the functions of the up-regulated genes. Interestingly however, many of these genes, especially genes up-regulated >5-fold, are located directly downstream and in the same orientation as various CRISPR loci (Fig 2A and Table 1). Predicted gene TTPX09 is located in a CRISPR locus, and is unlikely to encode a functional protein. Furthermore two putative transposases (TT_P0099 and TTC1169) of which expression appears up-regulated, are located near CRISPR loci on the genome, but in reverse orientation. TT_P0211 and TTPX12 are located directly downstream each other in a predicted operon. These and three other genes located on the chromosome (TTC0310, TTC0311 and TTC0399) appear to have no link with CRISPR loci. As we observe only elevated RNA levels under these conditions, it is highly unlikely that *Tt*Ago interferes with RNA, as this would lower RNA levels. Nevertheless, the fact that these genes are up-regulated only under conditions where both *Tt*Ago and plasmid DNA are present, suggests that *Tt*Ago directly or indirectly influences expression of these genes.

### Combined presence of plasmid DNA and *Tt*Ago results in up-regulation of crRNA expression

As many genes that are up-regulated in HB27+P are located on the genome near CRISPR loci, we further investigated expression of *cas* genes and CRISPR loci. Mega-plasmid pTT27 encodes complete Type I-C, III-A and III-B CRISPR-Cas systems (Fig 2A), and multiple scattered *cas* genes (two *cas1*, two *cas2*, one *cas4*, one *cas6* and a *cas3* gene with an internal frameshift (TTPX10 and TTPX11; Fig 2A and S1 Table). pTT27 additionally encodes eight CRISPR arrays (Fig 2). The HB27 chromosome encodes two *cas1* genes and a *cas6* gene, as well as two CRISPR loci (Fig 2). Besides TT_P0101 (encoding Cas2), no *cas* genes appear differentially expressed (S2 Table). This is striking, as the up-regulated *cas2* is located directly upstream of the predicted operon encoding the Type III-A CRISPR-Cas system (Fig 2A).

To investigate expression of CRISPR RNA (crRNA) from CRISPR loci, we used a dataset containing only reads that are partially complementary to CRISPR repeats (S4 Table). For most CRISPR loci, expression of crRNA is highest at the leader-proximal end of the CRISPR locus, and gradually lowered towards the leader-distal end of the CRISPR locus (S4 Table). This observation agrees with the leader harboring the promoter for crRNA expression [30,31]. When comparing crRNA expression in the different strains, expression of crRNAs encoded by eight CRISPR loci is strongly up-regulated in HB27+P compared to HB27 (Fig 2B and S4 Table). As some of the genes mentioned in Table 1 are located directly downstream CRISPR loci, it appears that the expression of these genes and the presence of a CRISPR locus is linked. This suggests that either these genes are expressed from the same promoter (read-through), or alternatively that they, and possibly other up-regulated genes that are not located directly downstream CRISPR loci, are under control of the same transcriptional regulator as the up-regulated CRISPR loci. As Cas2 and CRISPR leader sequences play essential roles in the acquisition of CRISPR-Cas-mediated immunity (reviewed in [30,31]), increased expression of Cas2 and crRNAs could imply that CRISPR adaptation is activated. To investigate if *Tt*Ago enhances CRISPR adaptation, we analyzed CRISPR loci for integration of new spacers. We used a PCR-based method that previously has been demonstrated to identify spacer integration in *E*. *coli* cultures, if at least 0.4% of the culture integrated a spacer in the amplified CRISPR locus [32]. However, no new spacers were detected, even when cultures were grown in the absence of antibiotics (S1 Fig). This suggests either that under the tested conditions CRISPR adaptation is not stimulated, or alternatively that CRISPR adaptation does not confer a benefit to the host (i.e. clones with novel spacers do not increase in frequency and therefore remain undetectable), which is supported by theoretical predictions that costly acquired immunity is not likely to evolve against parasites with low virulence [26].

### *Tt*Ago interferes with invader DNA but not with invader-encoded RNA

**Fig 3:**
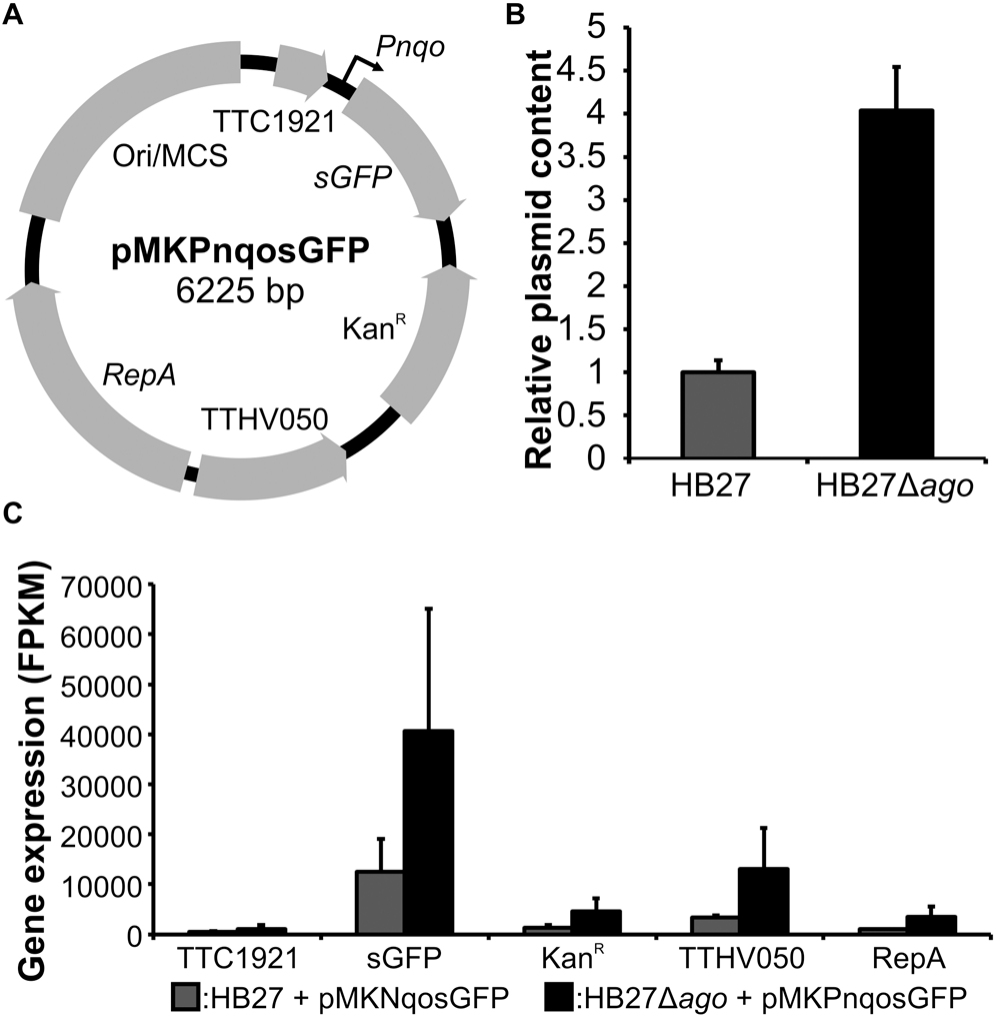
Effects of *Tt*Ago on plasmid DNA and plasmid encoded RNA. A, Schematic representation of the *Escherichia coli-T*. *thermophilus* shuttle vector pMKPnqosGFP. Ori/MCS indicates the *E*. *coli* origin of replication (Ori) and a multiple cloning site (MCS). Note that cloning of this plasmid resulted in insertion of (incomplete) TTC1921 and TTHV050 genes. B, Relative plasmid content of *T*. *thermophilus* strains HB27 and HB27Δ*ago* transformed with pMKPnqosGFP. Plasmid content was calculated from the complete DNA isolated from biological triplicates at an OD_600 nm_ of 0.5. C, Gene expression of plasmid encoded genes. Expression values are given in Fragments Per Kilobase of exon per Million fragments mapped (FPKM).

Besides the effect on genome-encoded gene expression, *Tt*Ago has a clear effect on plasmid DNA and plasmid-encoded gene expression (Fig 3 and S5 Table). It has previously been shown that *Tt*Ago interferes with intracellular plasmids, resulting in 3 to 5-fold higher plasmid contents in HB27Δ*ago* compared to HB27, even when the cultures were grown under conditions selecting for plasmid maintenance [12]. We determined plasmid pMKPnqosGFP content at the time at which the RNA was isolated (OD_600 nm_ of 0.5) for strains HB27+P and HB27Δ*ago*+P (Fig 3A and S5 Table). These cultures were grown in presence of kanamycin, selecting for pMKPnqosGFP maintenance. In line with previous observations [12], intracellular plasmid content was significantly (*P*<0.05) lowered ~4-fold in wild type HB27 compared to HB27Δ*ago* (Fig 3B), confirming that *Tt*Ago interferes with intracellular plasmid DNA. Furthermore, we observed 2.4 to 3.8-fold lower levels of plasmid-encoded RNA in the HB27+P strain compared to the HB27Δ*ago*+P strain (Fig 3C and S5 Table). Thus, in contrast to genomic encoded RNAs, plasmid encoded RNAs are lowered in the presence of *Tt*Ago. The ~4-fold lower plasmid content itself can explain the 2.4 to 3.8-fold decrease of plasmid encoded RNA, as there are less plasmid copies available for RNA expression. Thus, although DNA-guided *Tt*Ago has been shown to cleave both ssDNA and ssRNA targets *in vitro* [12–15], this data suggests that *in vivo Tt*Ago solely interferes with plasmid DNA and not with plasmid-encoded RNA.

Bacterial Ago from *Rhodobacter sphaeroides* (*Rs*Ago) associates with small RNAs and DNAs derived from extracellular sources such as plasmids, transposons and phages [11]. We therefore analyzed the effect of *Tt*Ago on RNAs encoded by transposases (S6 Table). We observed no higher expression of transposase genes in HB27Δ*ago* strains. In contrast, compared to HB27, in HB27+P levels of RNA mapped against predicted transposases TTC1169, TT_P0099 and TT_P0211 are significantly (*P*<0.02) increased (12.6-fold increase, 4.3-fold increase, and 6.6-fold increase, respectively). The fact that these differences were only observed if both *Tt*Ago and the plasmid are present, suggests that transposase expression is induced under these specific conditions only. As mentioned above, TTC1169 and TT_P0099 are located near CRISPR loci on the genome, suggesting their increased expression is a result of up-regulation of these CRISPR loci. As we only analyzed RNA levels, and because *Tt*Ago appears not to be interfering with RNA directly (see above), we cannot rule out that *Tt*Ago interferes with transposons at the DNA level (for example during the extrachromosomal step of their life-cycle).

## Conclusions

Only small stochastic changes in gene expression are observed when comparing wild type *Thermus thermophilus* and the derived Δ*ago* mutant. This implies that *Tt*Ago, in contrast to eAgos, is not involved in regulation of gene expression. In agreement with previous observations [12,17], *Tt*Ago lowers intracellular plasmid DNA levels, even under selective conditions for plasmid propagation. This results in decreased plasmid DNA levels that still allows for survival in the presence of kanamycin while lowering the metabolic burden of high copy number plasmids. Earlier work showed that *Tt*Ago preferentially acquires DNA guides complementary to plasmid DNA and/or plasmid-encoded RNA [12], and demonstrated that *Tt*Ago can cleave both DNA and RNA *in vitro* [12,15]. To investigate the effect of *Tt*Ago on plasmid DNA and on plasmid-encoded RNA, we analyzed new RNA-seq data derived from *T*. *thermophilus* strains harboring a plasmid. Compared to the Δ*ago* strain, we observed lowered plasmid DNA levels and accordingly lowered levels of plasmid-encoded RNA in the wild type strain. Strikingly, we observed no further reduction of plasmid-encoded RNA. This suggests that *Tt*Ago does not directly target RNA *in vivo*, making it a strict DNA-guided DNA-interfering host defense system.

Furthermore, the presence of plasmid DNA itself does not result in up-regulation of host defense genes. This suggests that, unlike phage infection, the presence of plasmid DNA is (at least under the used conditions) not registered as a threat. *T*. *thermophilus* requires a host defense system that is able to distinguish invader DNA from genomic DNA. While CRISPR-Cas systems require incorporation of spacers before being able to target invaders, *Tt*Ago specifically interferes with plasmid DNA without being dependent on genomic-encoded information about the invader. The observation that the combined presence of *Tt*Ago and plasmid DNA correlates with up-regulation of various CRISPR loci and at least part of the CRISPR adaptation machinery suggests that *Tt*Ago-mediated plasmid interference stimulates CRISPR adaptation. Although pAgos and CRISPR-Cas systems sometimes co-occur, often only one of these defense systems is encoded by a genome [9]. This suggests that these systems function independently. Nevertheless, there are rare examples where the gene encoding pAgo co-localizes with Cas1 and Cas2 (for example in *Methanopyrus kandleri*) or Cas4 (multiple pAgos) [9,10]. Cas1 and Cas2 are known to be essential for CRISPR adaptation [30,32,33]. Also Cas4 has been predicted to be involved in CRISPR adaptation as its forms complexes with Cas1 and Cas2 [34] and additionally Cas4 is fused to Cas1 in several Type I CRISPR-Cas systems [29]. As *cas* genes and pAgo do not strictly co-occur, we hypothesize that pAgo itself is not directly involved in spacer adaptation, but that pAgo-mediated plasmid interference indirectly stimulates CRISPR adaptation. For example, pAgos might generate plasmid DNA degradation products that somehow stimulate expression of genes involved in CRISPR adaptation. Acquisition of new spacers, stimulated by *Tt*Ago, would make future generations resistant against the invader by CRISPR-Cas-mediated defense. An additive effect of two host defense systems (a restriction modification system and a CRISPR-Cas systems) on total resistance levels has recently been reported [35]. Combined with observation that *Tt*Ago lowers plasmid concentrations even under conditions selecting for plasmid maintenance, this makes *Tt*Ago a valuable addition to the current arsenal of host defense systems.

## Materials and Methods

### Strains

*T*. *thermophilus* HB27 (ATCC BAA-163, DSM7039 and NBRC101085), which is referred to in this manuscript as HB27 or wild type, and the *Tt*Ago-encoding gene knockout strain HB27Δ*ago* [12] were used for the studies described in this manuscript (Fig 1).

### Transformations

*T*. *thermophilus* strains were transformed with plasmid pMKPnqosGFP [19] as described previously [12]. Colonies were selected and cultivated overnight at 65°C in 20 mL TTH medium [12] in a shaker incubator. 1 mL aliquots were prepared from the overnight cultures in 1.5 mL Eppendorf tubes which were centrifuged in a table top centrifuge at 6,000 rpm for 10 min. Supernatant was removed and cell pellets were stored at -20°C.

### RNA sequencing

*T*. *thermophilus* strains with and without plasmid pMKPnqosGFP were cultivated in triplicates as described previously [12]. Growth medium was supplemented with 30 μg/mL kanamycin for cultures harboring pMKPnqosGFP. When cultures reached an OD_600 nm_ of 0.5, RNA was purified using the mirVana RNA isolation kit (Ambion) as described previously [12]. Purified RNA from these biological triplicates was sequenced by BaseClear BV by Illumina sequencing.

### RNA-seq analysis

*T*. *thermophilus* genome was re-annotated using an in-house annotation pipeline SAPP platform (Koehorst *et al*., submitted). Reads of different experiments were all mapped against the *T*. *Thermophilus* genome (consisting of the HB27 chromosome and pTT27 mega-plasmid) and plasmid pMKPnqosGFP plasmid. For the identification of noise, reads of all experiments also excluding the pMKPnqosGFP plasmid were mapped against the entire *T*. *thermophilus* genome and corresponding plasmids. Differential expression analysis was performed using the trinity package in combination with RSEM [36].

### crRNA analysis

For the analysis of the crRNAs the CRISPR cassettes were predicted using the CRT prediction module in SAPP (Koehorst *et al*., submitted). The corresponding regions of the CRISPR cassettes were extracted and analyzed in combination with the gene sequences using the trinity package. To improve mapping, repeat regions were trimmed.

### Analysis of CRISPR loci

Triplicate HB27 and HB27Δ*ago* cultures with or without plasmid pMHPnqosGFP were cultivated in medium with and without antibiotics to an OD_600 nm_ of 0.5, after which genomic DNA was purified using the JGI ‘bacterial genomic DNA isolation using CTAB’ protocol [37]. Short stretches of each CRISPR locus, encompassing at least a part of the leader sequence and the first spacer-repeat unit, were PCR amplified (for primers see S7 Table), and resolved on 2% agarose gels. Gels were stained with SYBR Safe Nucleic Acid Stain (Invitrogen) and nucleic acids were visualized using a G:BOX Chemi imager. A comparable method has previously been demonstrated to detect CRISPR adaptation if at least 0.4% of the culture obtained new spacers [32].

### Plasmid content analysis

For complete DNA (containing both genomic and plasmid DNA) purification, *T*. *thermophilus* HB27 and HB27Δ*ago* transformed with pMKPnqosGFP were cultivated in triplicates to an OD_600 nm_ of 0.5. One OD_600 nm_ unit was harvested and complete DNA was isolated using the JGI ‘bacterial genomic DNA isolation using CTAB’ protocol [37]. 1 mg DNA of each purification was resolved on 0.8% agarose gels and stained with SYBR Safe Nucleic Acid Stain (Invitrogen), visualized using a G:BOX Chemi imager and analyzed using GeneTools analysis software (Syngene).

### Statistical analysis

For the calculation of *P* values of differences in expression levels of specific genes, FPKM of biological triplicates of each strain were used as the input. *P* values stated in this manuscript are calculated by a two-tailed distributed two-sample t-test assuming equal variances.

## Supporting Information

S1 Fig PCR of CRISPR loci. 1–3: HB27 grown in absence of antibiotics. 4–6: HB27+P grown in absence of antibiotics. 7–9: HB27+P grown in presence of antibiotics. 10–12: HB27Δ*ago* grown in absence of antibiotics. 13–15: HB27Δ*ago*+P grown in absence of antibiotics. 16–18: HB27Δ*ago* grown in presence of antibiotics. M: GeneRuler 100 bp plus DNA ladder (Thermo Scientific). Black triangles indicate expected sizes of PCR products if no new spacer are acquired. If new spacers are acquired a new band ~75 bp larger than the original band is expected. No spacer acquisition was observed.
(TIF)

S1 Table FPKM of the RNA-seq datasets and newly identified genes. (XLSX)

S2 Table Fold-change in FPKM for various strains. (XLSX)

S3 Table Comparison of differentially expressed genes in *T*. *thermophilus* strains HB8 and HB27 *ago* knockout strains. (XLSX)

S4 Table CRISPR RNA expression. (XLSX)

S5 Table Plasmid DNA content and plasmid RNA expression. (XLSX)

S6 Table Transposase RNA expression. (XLSX)

S7 Table Primers used for PCR amplification of T. thermophilus HB27 CRISPR loci. (XLSX)
