# Supplementary figures and images for: Effects of Argonaute on Gene Expression in Thermus thermophilus
Source: PLoS One. 2015 Apr 22;10(4):e0124880. doi: 10.1371/journal.pone.0124880 (PMC4406477; doi:10.1371/journal.pone.0124880)

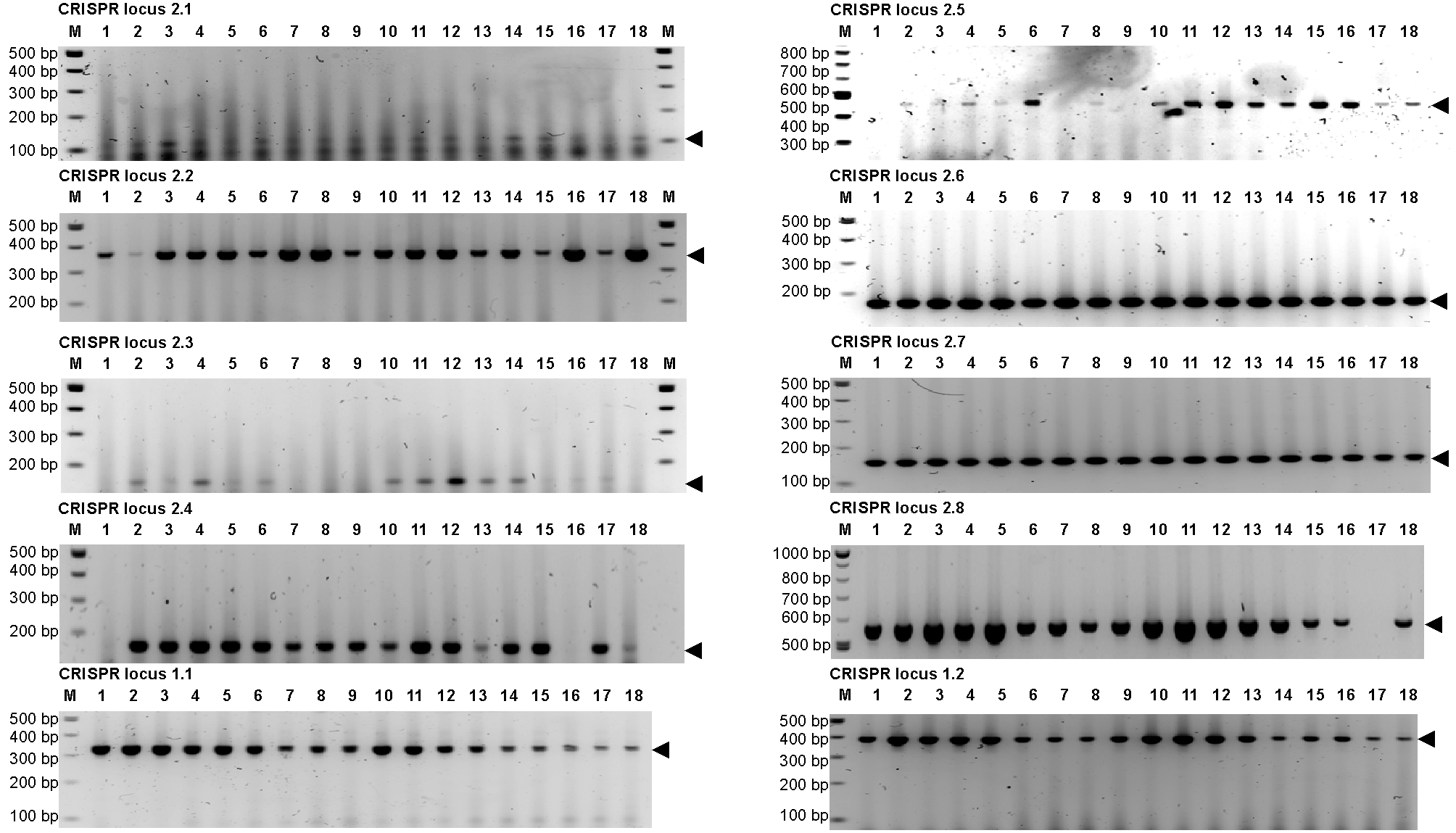

Supplement: S1 Fig — 1–3: HB27 grown in absence of antibiotics. 4–6: HB27+P grown in absence of antibiotics. 7–9: HB27+P grown in presence of antibiotics. 10–12: HB27Δago grown in absence of antibiotics. 13–15: HB27Δago+P grown in absence of antibiotics. 16–18: HB27Δago grown in presence of antibiotics. M: GeneRuler 100 bp plus DNA ladder (Thermo Scientific). Black triangles indicate expected sizes of PCR products if no new spacer are acquired. If new spacers are acquired a new band ~75 bp larger than the original band is expected. No spacer acquisition was observed. (TIF) [file pone.0124880.s001.tif]
